# Future Perspectives and New “Frontiers” in Cardiac Rhythmology

**DOI:** 10.3389/fcvm.2020.00126

**Published:** 2020-08-26

**Authors:** Matteo Anselmino, Gaetano Maria De Ferrari

**Affiliations:** Division of Cardiology, Department of Medical Sciences, “Città Della Salute e Della Scienza di Torino” Hospital, University of Turin, Turin, Italy

**Keywords:** arrhythmias, electrophysiology, technologies, innovation, cardiac

## Abstract

In the last three decades the Cardiac Rhythmology field has experienced tremendous change and evolution. Our understanding of the underlying mechanism of arrhythmic diseases has dramatically improved, starting from the genetic and molecular mechanisms. Innovative pharmacological and non-pharmacological treatment options have been introduced, and arrhythmias previously considered “untreatable” are now successfully managed in most referral centers. The increasing awareness of the detrimental effects of arrhythmias on any underlying cardiac substrate, targeted as a potentially modifiable cause, has therefore led to an increasingly stronger effort in developing novel methods and approaches to treat arrhythmia and improve patients' health and quality of life. Of all potentially significant developments in the field, we have decided to focus on the approaches generally applicable to multiple arrhythmic cardiac disorders and related to the advancement of technology. More specifically, we will deal with electroanatomical mapping and lesion creation during interventional procedures.

## Fluoroscopic Imaging, Ages Ago

Drug treatment is no longer the sole option for management of complex arrhythmia substrates, such as those underlying atypical atrial flutter or ventricular tachycardia. Transcatheter ablation will increasingly become the preferred treatment option. However, extensive ablative procedures during which catheters are navigated in the heart by fluoroscopic visualization are exposed to high X-ray doses. Driven by the considerable evidence documenting the hazards of this exposure to both patients and medical personnel (1, 2), alternative options have emerged. Three-dimensional (3D) electroanatomical mapping systems permit visualization of the catheters and of the created lesions in non-fluoroscopic virtual views, allowing near-zero radiation procedures (3–5) which may become the gold-standard for many approaches.

These systems, originally designed to offer precise electrical activation and voltage data, have improved substrate recognition, significantly advancing treatment of complex arrhythmias. In addition, by integrating with fluoroscopy, cardiac tomography (CT), magnetic resonance (MR), and intravascular echography imaging, they have enabled interventional approaches to highly complex cardiac anatomies. The three most widely used mapping systems (EnSite NavX by Abbott; Carto by Biosense Webster; and Rhythmia by Boston Scientific) will continue to improve and will be accompanied by new options with the potential to further implement cardiac substrate analysis, without exposure to harmful radiations.

### Cardiovascular Magnetic Resonance

Cardiovascular magnetic resonance (CMR) has experienced fantastic advancements. Compared to fluoroscopic imaging it permits superior cardiovascular anatomy delineation and advanced multiplanar reconstruction. In addition, a unique and futuristic feature is the ability to provide physiologic information of the sampled target (6). CMR may quantify, by velocity-encoded phase-contrast, blood flow in the major vessels, accurately determining cardiac output and pulmonary-to-systemic flow ratio (7). By cine steady-state free precession, global, and regional ventricular function can be assessed (8). Recently, CMR has been tested to guide electrophysiological procedures.

The are several potential benefits related to a CMR suite. Arrhythmia substrate, as areas of tissue damage or scar, can easily be characterized (e.g., concerning depth: epicardial, mesocardial, endocardial, or transmural), facilitating localization of the likely ablation target area and suggesting ideal procedural approach (e.g., endocardial vs. epicardial) (9). The clear visualization, on a multiplanar level, of all structures contiguous to the heart, especially in complex cardiac anatomies, may improve procedural safety, limiting damage to structures not trackable by fluoroscopy (e.g., phrenic nerve, esophagus). Last, lesions created by ablation can be seen directly, offering the unique possibility to provide intraprocedural objective assessment of ablation's effect.

Crucial to the success of interventional CMR is real-time tracking and visualization of catheters and guidewires in this environment. Thanks to improved machine processing power and faster image acquisition and reconstruction algorithms, it is now possible to achieve frame rates up to 20 images/s, with a spatial resolution suitable for interventional applications (10). Out of more than a few, centers in London (UK) (11) and Leipzig (Germany) (12) are, to date, the most active, routinely performing procedures and gathering sizable experience. Toupin et al. in Bordeaux, have demonstrated technical feasibility of real-time, *in vivo*, direct assessment of radiofrequency lesion formation in the myocardium, with a precision in the range of 1 mm (13). Imaging of the ablation lesions during energy application presents the invaluable potential to enable real-time energy titration to the exact amount, ensuring transmurality.

However, limitations exist. The cost associated with installing expensive CMR suites limits widespread application of this technology, although the approach may prove to be cost-effective if associated to a significant improvement in patient outcome. In addition, patients with non-MR compatible implanted cardiac devices would not be suitable for treatment and those with MR conditional devices suffer from artifacts obscuring the underlying anatomy. Eventually, despite several tracking methods being available (14), interventional tools suitable for CMR environment are limited. The ideal tracking catheter or guidewire should not include ferromagnetic materials which cause large susceptibility artifacts, but be constructed of alloys, such as nitinol, that permit adequate physical properties (e.g., torque and steerable).

### New Electroanatomical Mapping Systems

**Figure 1:**
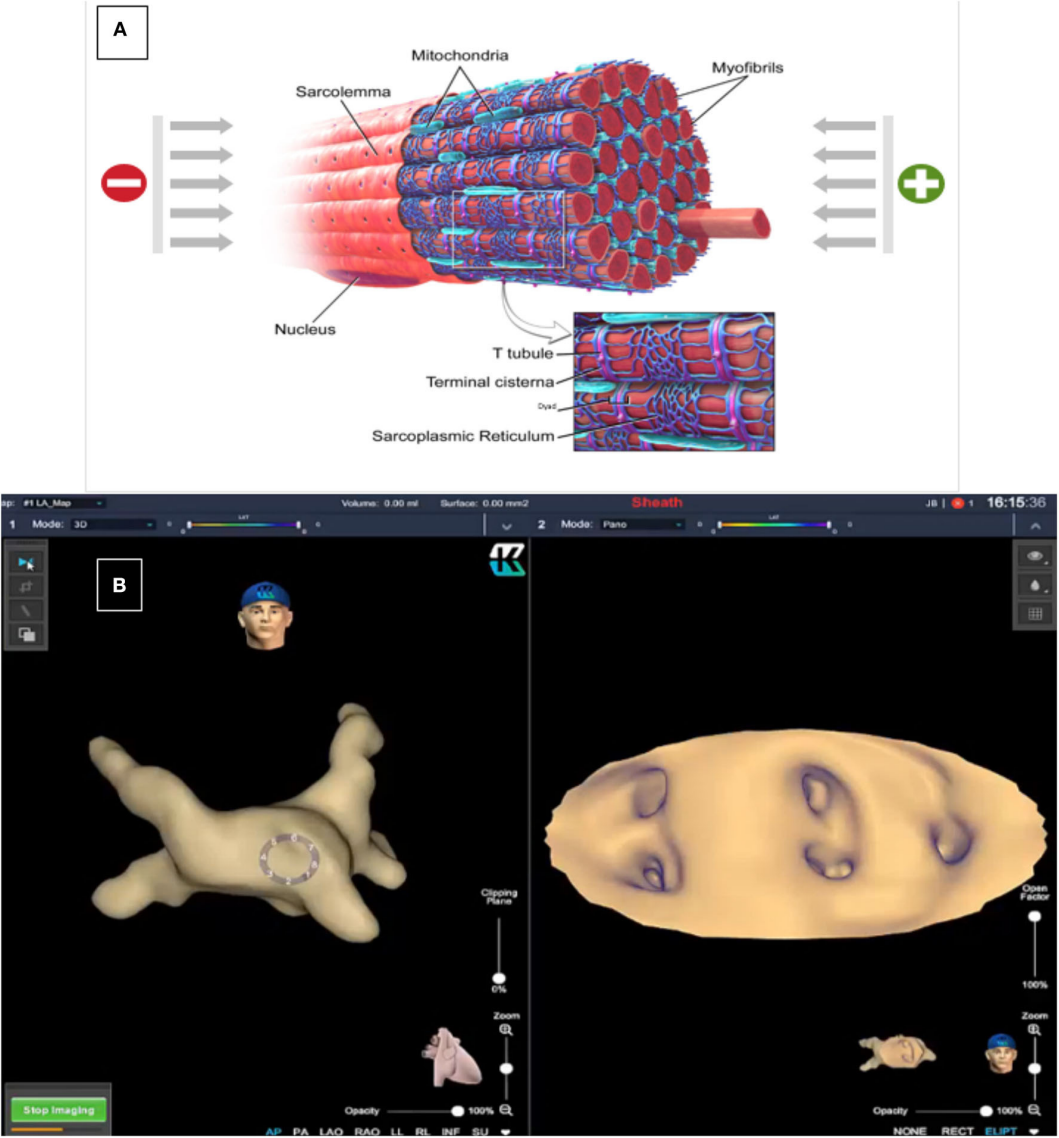
Dielectric Sensing Technology has the ability to differentiate material characteristics by measuring their dielectric coefficients. In the myocardium, electric charges can be conducted directly through the tissue (mainly through the intracellular fluid) but part of the charges will be stored (mainly in the sarcoplasmic reticulum that serves as a capacitor). These charges will be released after a short time. The conduction and capacitance values are frequency dependent and are affected by the state of the tissue **(A)**. This technology permits 3-D reconstructions and CT-like endocardial views of the different cardiac tissues [**(B)**, left: 3-D reconstruction of the left atrium; right: endocardial view of the four pulmonary veins and left atrial appendage ostia]. Image courtesy of EPD Solutions, a Philips company.

In addition to the continuous improvement of the available mapping systems with new algorithms, novel tools have recently been launched. The KODEX-EPD (Philips) is a new, non-fluoroscopic, dielectric-based imaging, and navigation system (15). Through fluctuating voltage measurements within an electric field, this system enables 3D real-time visualization of any electrode-containing catheter. The internal (catheter) and external (body surface patches) electrodes transmit and receive low-amplitude frequencies and phased electrical signals, permitting to measure electromagnetic properties of the sampled structures. The derived electromagnetic signature permits to compute location and contact pressure. Marked gradients in the electrical field occur near borders of the different cardiovascular structures, as the endocardial surface, vessels, or valves, and this “bending of the field” is sensed by the system, and represented as a boundary on the 3D map. By assigning a set of electrical field descriptors to each location visited by the catheter, a 3D map is created, and, thanks to the derived electromagnetic signature of the different interfaces, contact pressure is computed. On the virtual map, by comparing voltage differences, the system may apply an “internal ruler,” providing measurable distances. In fact, KODEX-EPD generates high-resolution 3D cardiac anatomical images, without the need for direct catheter-tissue contact or pre acquired imaging, at a spatial resolution of ~0.3 mm (16) (Figure 1). The scans of the sampled cardiovascular structures achieve such fine details (e.g., structure's thickness) that may support the decision on the amount of energy to titrate, and aid in the validation of lesion formation (e.g., identification of ablation gaps). Notably, unlike other mapping systems that employ vendor-specific catheters, this technology potentially operates with virtually any intracardiac mapping/ablation catheter/electrode device and only requires disposable body surface patches. Thus, although additional research is needed to assess reliability, KODEX-EPD holds the potential to represent an open-platform that allows high quality procedures at reduced cost and burden on the patient. The system could allow most electrophysiology centers to generate high-definition 3D non-fluoroscopic images without the need for prior cardiac CT or MR scans, while continuing using their routine (electrode-containing) catheters (17).

A second recently introduced system is the AcQMap (Acutus Medical), a high-resolution imaging and mapping system that combines ultrasound-guided anatomical views with bipolar/unipolar signals registered with a specifically designed catheter. The latter consists of a hybrid spheroid with six splines, each populated with eight ultrasound transducers and eight biopotential electrodes, adding up to 48 ultrasonic transducers and an equal number of electrodes. The ultrasound transducers sample the cardiac chamber at a rate of 115,000 points/min, to rapidly create an anatomic reconstruction; the biopotential electrodes collect up to 150,000 samples/second, to generate a 3D, real-time, electrical activation map. Electrical data collection of this system is extremely innovative. The algorithm processes multiple non-contact voltage measurements, showing a charge-density source distribution across the endocardial surface. The charge-density approach, based on the first principles of electrostatic field theory, has shown to perform well, in terms of both spatial and temporal metrics, reporting a median error of about 1.7 mm and <0.96 ms, respectively, compared to established computer-simulated models of cardiac conduction (18). If the preliminary encouraging findings will be confirmed, AcQMap may represent a revolution of the fundamental basis guiding electrophysiological mapping in the last 50 years.

## Once Upon a Time Unipolar Radiofrequency Ablation…

Since its introduction in 1987, radiofrequency catheter ablation has become an established option in the management of patients with arrhythmias. Relapses, however, unfortunately still occur and complete success in all patients is far out of reach. One of the reasons for disappointment is the failure to create transmural lesions. Also, in the presence of a consolidated target such as pulmonary vein isolation for the treatment of atrial fibrillation, achievement of the desired goal has proven challenging. In a large US series, at the time of a third or more ablation, re-connection of at least one pulmonary vein was documented in up to 90% of the patients (19). In fact, when radiofrequency ablation is performed by a point-by-point approach, it is strongly operator-dependent and gaps within the applied lesions are seen quite often. Recently, new lesion quality markers have been introduced but their clinical validation is ongoing (20). For this reason, for standard clinical applications such as atrial fibrillation, several one-shot techniques, simplifying and standardizing lesion outcomes, have proved successful (21, 22).

In complex settings such as ventricular arrhythmias due to ischemic heart disease or cardiomyopathies, which require individual identification of the ablation target that may be hidden deep within the myocardium, the risk of not achieving permanent transmural lesions certainly contributes to a reduced success rate.

### Bipolar Radiofrequency Ablation

A better understanding of the biophysical properties of radiofrequency energy has driven important innovations, translating into the hope of higher rates of success, and similar, if not improved, safety profiles, compared to the traditional approach.

Standard radiofrequency ablation is performed by irrigated-tip catheters in a unipolar configuration. The catheter tip acts as the active electrode, and a ground patch, usually placed on the patient's back or leg, as the reference electrode. Arrhythmia substrate depends on the underlying condition; just as an example at ventricular level an ischemic disease more commonly implies an endocardial, while non-ischemic and infective etiologies, an epicardial substrate. Thickness of the ventricular wall or presence of epicardial fat may therefore prevent reaching the ablation target with standard unipolar ablation. Differently, bipolar ablation focuses radiofrequency energy between the closely placed distal electrodes of two ablation catheters, one being the active electrode, and, the second, the return patch. By this configuration, the applied energy produces a rapid rise in tissue temperature and consequent thermal injury (23). Due to this distinctive mechanism of lesion formation, bipolar ablation represents an appealing alternative for creating permanent transmural lesions, especially in deep arrhythmia substrates, as left ventricle papillary muscles, interventricular septum, left ventricular mid-myocardium, or fat-insulated epicardium (24, 25).

A recent animal study was designed to compare last generation contact force-assisted bipolar radiofrequency ablation against sequential unipolar approach to test superiority in terms of creating deep and transmural lesions, from both the endocardial and epicardial site. Swines were selected for their similarity in anatomy and organ size to humans. Lesions from the endocardial and the epicardial side of the ventricular free walls were analyzed by non-destructive high-resolution 9.4T magnetic resonance imaging, providing precise localization, linear, and volumetric assessment. With a fixed 30 Watts and 60 s ablation set, achievement of a transmural lesion was more common by bipolar ablation (OR 23.73, *p* = 0.030) (26). Additionally, bipolar radiofrequency ablation holds the potential of limiting damage to non-cardiac structures contiguous to the heart (e.g., phrenic nerve, pericardium, lungs), focusing resistive heating in a more localized area compared to the traditional unipolar approach. On the other side, excessive focused heating may create steam cavitation, the so-called “pop” that may cause serious adverse events. Although the first experiences have not reported major complications while ablating in bipolar mode, power, time, and contact force settings still require careful tuning before implementing its routine use.

### Electroporation

**Figure 2:**
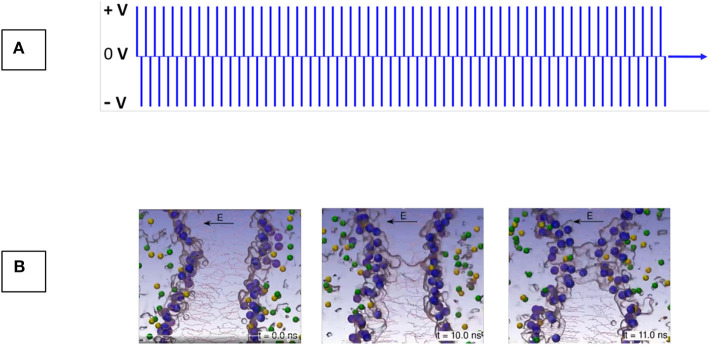
Irreversible Electroporation (IRE) is the mechanism of killing tissue through exposure to high electric field gradients **(A)** that induce permanent, hyper-permeablization of the cell membranes. Permeabilization of the lipid bilayer begins within nanoseconds after application of sufficient electric field gradients **(B)**. Images courtesy of Medtronic and Prof. Damijan Miklavčič, University of Ljubljana, Slovenia.

A common limitation, with the classical ablation sources, is the inability to apply energy on the ablation target area in case of proximity to sensible non-cardiac structures (e.g., phrenic nerve, esophagus, blood vessels). For this reason, electroporation, an entirely novel, unique tissue-selective ablation method, appears an extremely attractive tool in the future management of arrhythmias. By localized direct-pulsed electric exposure, this technology increases cell membrane permeability in a cell-specific manner. While reversible electroporation has been studied since the 1980s (27), the concept of an irreversible effect of electroporation is rather new and has just recently been explored for cardiac applications (28). In fact, among cell types, cardiomyocytes have amongst the lowest thresholds to these fields, potentially permitting preferential myocardial ablation (29). In addition, application of different thresholds of currents may permit multifaceted cell-specific modulation, predicting effects on cardiac myocardium, cardiac conduction tissue, or non-cardiac tissue (Figure 2). An animal study performed at Mayo Clinic assessed whether this technology may permit to selectively target the cardiac Purkinje system (30). By applying, in a bipolar fashion, currents at voltages between 500–3,500V at a frequency of 0.83–1 Hz with a pulse duration of 20–90 ms, they demonstrated feasibility of an acute dose-dependent functional effect on the Purkinje system. Histopathological analysis confirmed acute Purkinje fiber targeting, with minimal subendocardial myocardial fibrosis. The possibility to target a specific subtype of cells, as in this experience, without injuring the surrounding myocardial tissue, appears very appealing. The only currently acknowledged risk is arrhythmia induction; to mitigate this risk, pulses need to be synchronized and delivered during the absolute refractory period. However, safety and efficacy in adequately sized human series still need to be established.

### Stereotactic Radioablation

In the setting of complex anatomies (congenital heart disease or post cardiac surgery) and advanced arrhythmia substrates, challenging ablation target identification and reachability may easily explain the high recurrence rates reported by conventional approaches (31). In addition, repeat, and increasingly invasive (e.g., epicardial access, high power settings) procedures unfortunately increase the risk of complications, which is also dreadful. In this scenario, there is compelling need to develop safer, more effective therapies.

A new treatment modality has been proposed with promising early results (32). Stereotactic radioablation is a non-invasive and rapid (in the order of 15–30 min) method to deliver photons (X-rays), protons, or heavy ions (carbon) into selected cardiac tissue. Based on a close collaboration between radiotherapists and electrophysiologists, a single high intensity radiation dose, by respiratory and cardiac gating, can be selectively delivered to a small volume of the heart.

Robinson et al. (33) have presented a prospective study evaluating stereotactic body radiation therapy on 19 patients. Following a 25 Gy radiation directed to a target defined by non-invasive electroanatomic mapping (multielectrode surface ECG-based mapping), several measures of ventricular arrhythmia burden reduction remarkably reached significance. As an example, the reported median number of arrhythmia episodes decreased from 119 (4–292) to 3 (0–31) in the 6 months before, and after the ablation, respectively. Several adverse events were observed, with two, pericarditis and radiation pneumonitis, directly related to the treatment. One year survival reflected the comorbidities present in the enrolled population (72%), however, three of the deaths were associated with recurrent arrhythmia during worsening heart failure.

In Czech Republic, the group of Neuwirth et al. reported longer term follow-up data (34). Ten patients exposed to a single 25 Gy exposure, delivered to a target identified with traditional electroanatomical mapping, achieved, after a median of 28 months, an arrhythmia burden reduction of 87%. Also in this experience, the safety profile appeared satisfactory: four patients experienced nausea and one patient presented gradual progression of mitral regurgitation, while three patients suffered non-arrhythmic deaths.

Recently, a different US group reported, in a 6-month follow-up period after a single fraction 25 Gy dose, a reduction in implantable defibrillator shocks of 65%. Two patients experienced mild pneumonitis, responsive to steroid therapy, and one patient suffered acute arrhythmia induction requiring cardiac resuscitation. A peculiarity of this study is availability of cardiac histology on three patients that received heart transplant after the treatment. Based on a preliminary microscopic analysis, treated regions indicated edema, and vacuolization of endothelial cells with mild fibrosis, in one case disruption of intercalated disc/gap junction areas, which may explain the relatively acute treatment responses (35).

Several challenges remain open. Not all centers report similarly positive results. In the study by Gianni et al. for example, five patients underwent 25 Gy single fraction radioablation delivered by CyberKnife, and all patients experienced clinically significant mid- to late-term ventricular arrhythmia recurrence (median follow-up of about 1 year) (36). In addition, to date, the ability for simultaneous gating of respiratory and cardiac movement is lacking. In this respect, the recent first in men stereotactic radioablation performed on a hybrid MR-Linac field permitting real-time tracking and beam-gating is, indeed, promising (37).

Indeed, clear comprehension of the mechanisms involved, and the question whether protons or heavy ions may provide a benefit compared to photons (38) require answers. However, this innovative approach may already be considered as a bailout strategy and holds great potential for the future.

## Final Thoughts

The Cardiac Rhythmology field is running quick. Improvements in electroanatomical mapping and lesion creation will improve our efficacy in treating patients, independently of the type of structural substrate.

Other innovative approaches, however, appear ready for a formidable expansion. Optogenetics is an attempt to control and monitor the biological functions of a cell, group of cells, tissue, by using optical systems and genetic engineering technologies (39). Stem cell-based therapies aim to regenerate damaged cardiac muscle by replacing dead tissue with new contracting myocytes or replace misfunctioning ion channels (40) and virtual electrophysiology to model bioelectronic heart-on-a-chip models for studying the effects of different noxa on cardiac function (41). These futuristic approaches may shift the goal further up, aiming to prevent, silence or freeze the underlying electrical substrate or cardiomiopathy before it even causes an arrhythmia.

## Author Contributions

MA and GD drafted and designed the manuscript. All authors contributed to the article and approved the submitted version.

## Conflict of Interest

The authors declare that the research was conducted in the absence of any commercial or financial relationships that could be construed as a potential conflict of interest.
